# Toward the Bayesian brain: a generative model of information transmission by vestibular sensory neurons

**DOI:** 10.3389/fneur.2024.1465211

**Published:** 2024-12-18

**Authors:** Michael G. Paulin, Kiri F. Pullar, Larry F. Hoffman

**Affiliations:** ^1^Department of Zoology, University of Otago, Dunedin, New Zealand; ^2^Department of Head and Neck Surgery and Brain Research Institute, David Geffen School of Medicine at UCLA, Los Angeles, CA, United States

**Keywords:** hair cell receptor, spike train analysis, neural coding, stochastic point process, computational neural model, perception as Bayesian inference, cerebellum, cerebellar-like

## Abstract

The relative accessibility and simplicity of vestibular sensing and vestibular-driven control of head and eye movements has made the vestibular system an attractive subject to experimenters and theoreticians interested in developing realistic quantitative models of how brains gather and interpret sense data and use it to guide behavior. Head stabilization and eye counter-rotation driven by vestibular sensory input in response to rotational perturbations represent natural, ecologically important behaviors that can be reproduced in the laboratory and analyzed using relatively simple mathematical models. Models drawn from dynamical systems and control theory have previously been used to analyze the behavior of vestibular sensory neurons. In the Bayesian framework, which is becoming widely used in cognitive science, vestibular sense data must be modeled as random samples drawn from probability distributions whose parameters are kinematic state variables of the head. We show that Exwald distributions are accurate models of spontaneous interspike interval distributions in spike trains recoded from chinchilla semicircular canal afferent neurons. Each interval in an Exwald distribution is the sum of an interval drawn from an Exponential distribution and a Wald or Inverse Gaussian distribution. We show that this abstract model can be realized using simple physical mechanisms and re-parameterized in terms of the relevant kinematic state variables of the head. This model predicts and explains statistical and dynamical properties of semicircular canal afferent neurons in a novel way. It provides an empirical foundation for realistic Bayesian models of neural computation in the brain that underlie the perception of head motion and the control of head and eye movements.

## 1 Introduction

Mathematical modeling of the vestibular system was pioneered by Steinhausen (1) [reviewed by Straka et al. (2)], who developed a differential equation model of the mechanics of transduction in the semicircular canals during head movement. This equation, which became known as the torsion pendulum model, provided a foundation for later dynamical models of the sense organ, afferent neurons, central neural circuitry, and head and eye stabilizing reflexes during head rotation (3). Researchers showed that torsion pendulum-like dynamical models can predict firing rates of semicircular canal afferent neurons during head rotation in species drawn from all vertebrate orders from fish to mammals. However, they also discovered unexpectedly high variability in response properties and correlations between parameters of models fitted to different neurons (3). These discoveries led to population rate-coding models, in which the idiosyncratic behavior of individual neurons is quantified by differential equations or equivalent transfer function models that predict firing rate (4–9). Stochasticity was modeled by a descriptive statistic, CV^*^, which measures the relative variability of spontaneous interspike intervals (10).

Analysis of model parameters revealed that dynamical response properties co-vary systematically with each other and with CV^*^ across the afferent population. The same pattern is found in all species. Units with more regular spontaneous firing activity (low CV^*^) tend to have higher spontaneous and average firing rates, to be less sensitive to rotation, and to fire in phase with angular velocity (11). Units with more irregular spontaneous firing activity (high CV^*^) tend to have lower spontaneous and average firing rates, to be more sensitive to rotation, and to be phase-advanced relative to angular velocity. Parameters vary continuously between these two extremes. Goldberg and Fernandez (10) introduced the terms “regular” and “irregular” as a “rhetorical convenience” associated with simple bivariate statistical analysis on artificially defined subsets of the data. This has led to a persistent misconception that there are two naturally occurring functional subclasses of neurons in the afferent nerve (3, 12).

These models could describe the data but did not explain why the neurons behave as they do or why the patterns occur. We suggest approaching such questions by considering models of behaviors that depend on information provided by vestibular sensory neurons. Borah et al. (13) developed a model of head-eye coordination in the framework of stochastic optimal control theory. A stochastic optimal controller decomposes mathematically into two components in series. The first component, called a state estimator or observer, estimates the relevant state variables from noisy data. The second component uses the state estimate to compute an optimal control signal, taking into account the observer's uncertainty about the true state [reviewed by (14)]. Optimal stochastic control models were able to predict dynamical and psychophysical features not predicted by earlier deterministic models developed in the framework of classical control theory. This suggests not only that the brain takes account of uncertainty in sense data when it computes control signals but also that head-eye coordination may involve two stages of neural information processing. In the first stage the brain uses sense data to construct a belief about (i.e., perceive) relevant world and body states, and in a second stage the brain decides how to respond given what it perceives, i.e., how to act given what it believes. The prediction that the vestibular-cerebellar hindbrain contains (or is) a neural analog of a dynamical state estimator is supported by a range of anatomical, behavioral and psychophysical evidence (15).

Despite its success in modeling the dynamics of head-eye coordination, stochastic optimal control theory has had little impact on models of underlying neural mechanisms, no doubt partly because the theory is restricted to systems with linear dynamics and Gaussian errors, and employs an algebraic formalism that cannot be mapped onto neural mechanisms in a realistic way (16, 17). But linear-Gaussian stochastic optimal control theory is now recognized as a special case of Bayesian decision and control theory (18, 19), which has become a standard modeling framework in cognitive science (20, 21). A Bayesian observer computes the conditional probability distribution of states, a.k.a. the posterior distribution, given some measurements. In the special case of linear dynamical systems with Gaussian measurement errors the mean and covariance of this distribution, i.e., the entire distribution in that case, can be calculated algebraically by an algorithm called the Kalman filter (22).

The success of Kalman filter models at the psychophysical and behavioral level (23) suggests that a way forward at the neural level is to model semicircular canal afferent neuron spike trains as observations transmitted to a Bayesian observer in the brain, without assuming linear dynamics or Gaussian uncertainties. In the Bayesian framework, observations are random samples from probability distributions parameterized by variables of interest to the observer. It follows that the first task in constructing or analyzing a Bayesian observer is to determine the probability distribution of the data parameterized by the relevant variables.

Existing models show robustly that the relevant state variables for modeling information transmission by semicircular canal afferent neurons are angular velocity and angular acceleration around the canal axis (3). Therefore, we propose to model semicircular canal afferent neuron spike trains as sequences of intervals, each of which is a random sample from a probability distribution whose shape depends on the kinematic state of the head. This turns the conventional approach on its head, making quantification of stochasticity the primary goal of modeling, rather than merely an accounting procedure that quantifies the difference between models and data and explains it by calling it noise.

In this paper we identify probabilistic models of spontaneous firing using spike train data recorded from semicircular canal afferent neurons in chinchillas. At first sight, models of spontaneous activity, which describe how the neurons behave when the head is not moving, would seem to have limited value for understanding neural mechanisms of sensory-motor control when the head is moving. However, holding the head steady is an ecologically important behavior for many animals, for example while attending to auditory and visual cues that may betray a nearby predator or prey. Optimal control of this behavior requires inferring the kinematic state of the head from measurements when the true state is near the goal state, i.e., when the head is nearly stationary. Intuitively, a model that describes the behavior of a stochastic dynamical system in a particular state should be continuously modifiable to describe the system's behavior in nearby states. In that case a model of spontaneous activity may be extended to provide a model of driven activity in a small region of head kinematic state space relevant to explaining ecologically important behavior.

The potential for a model of spontaneous activity to generalize to model driven activity is presaged by the ability of CV^*^, a simple summary statistic of variability in spontaneous activity, to predict the dynamical behavior of semicircular canal afferent neurons during head movement. After presenting our probabilistic model of spontaneous firing we will expand on this point and discuss how our model may be used to analyze neural mechanisms underlying statistically optimal control and coordination of head and eye movements. This provides a simple accessible model system for empirically grounded, realistic analysis of how brains use sense data to perceive relevant world and body states, and how brains make decisions and control actions based on data-driven internal beliefs about relevant states and parameters.

## 2 Materials and methods

### 2.1 Spike train data acquisition

All procedures involving animals were approved by the UCLA Chancellor's Animal Research Committee and conformed to guidelines mandated in the *NIH Guide for the Care and Use of Laboratory Animals* (National Institutes of Health Publication, revised 2011), and the *Guidelines for the Use of Animals in Neuroscience Research* (Society for Neuroscience).

#### 2.1.1 Animal preparation

Adult male chinchillas (*n* = 27; body mass 450–650 grams) were used in these experiments. They were first anesthetized with isoflurane, after which an intravenous cannula was secured within a jugular vein through which maintenance doses of sodium pentobarbital (0.05 cc, 50 mg/cc) were administered. A tracheotomy was performed into which a catheter delivering 100% O_2_ was loosely placed. Heart and respiratory rates, as well as O_2_ saturation levels, were monitored throughout the surgical preparation and recording session. Core body temperature was maintained between 38°-38.5°C with a custom servo-controlled heater and rectal thermocouple probe. Animals remained physiologically stable throughout the long electrophysiologic recording sessions, which at times lasted longer than 12 h.

Upon achieving a surgical plane of anesthesia animals were fit into a custom head holder fixed to a turntable. Surgical procedures were similar to those utilized in previous investigations of vestibular afferent electrophysiology (24). The right middle ear was exposed by removing the bony cap of the tympanic bulla. The bony ampullae of the superior and horizontal semicircular canals were identified, which provided landmarks to the internal vestibular meatus channeling the superior vestibular nerve between the labyrinth and brainstem. The superior vestibular nerve was exposed at this site, approximately 1–2 mm from the landmark ampullae, using fine diamond dental drill bits. Final exposure of the nerve was achieved by gently teasing the epineurium from the nerve with electrolytically sharpened pins.

#### 2.1.2 Single afferent electrophysiology

Spontaneous discharge epochs from 330 semicircular afferents within the superior vestibular nerve were recorded with high-impedance microelectrodes (40–60 MΩ) driven by a piezoelectric microdrive. Spontaneous discharge was detected as the electrode approached an afferent, and generally improved with subtle adjustments in electrode position achieved by small manipulations of the microdrive (e.g., small forward and reverse displacements, in addition to gentle tapping of the drive). Upon achieving stable recording, manual turntable displacements were used to identify the epithelium from which the afferent projected. Afferents innervating the horizontal and superior cristae increased their discharge to rotations resulting in utriculofugal and utriculopetal endolymph flow, respectively, and would decrease in discharge in response to turntable rotations in the opposite direction. Afferents projecting to the utricle were generally unresponsive to rotations, or increased their discharge during application of rotations in both directions (centripetal displacements of the otolithic membrane concomitant with rotation in either direction). These afferents were excluded from the present dataset.

### 2.2 Data analysis

#### 2.2.1 Data acquisition, summary statistics and exploratory analysis

Single-unit spike times were acquired in 20-second records with 300μ*s* resolution, and imported into MATLAB as arrays of interspike interval (ISI) lengths in seconds. Plots of spike time data and ISIs were visually inspected to identify trends, discontinuities and outliers indicating possible miss-triggering during data acquisition. We tested for serial correlation in interval length using a Wald-Wolfowitz runs test (MATLAB function runstest). Records with detectable artifacts or non-stationarity were removed, leaving 306 of an initial 330 records for further analysis and modeling.

Mean ($\bar{x}$), standard deviation (*s*), coefficient of variation ($CV=s/\bar{x}$) and Pearson's moment of skewness (γ = *E*[(*x*−μ)^3^]/σ^3^) were computed for the intervals in each spike train, using MATLAB functions *mean, std*, and *skewness*. Standard deviations of interval length for the most regular units in our sample are comparable to the resolution of spike time data acquisition (300μ*s*). Because of this, estimates of CV and skewness for very regular units may be less reliable than estimates for irregular units. CV is a scale-invariant measure of variability. It is near zero for highly regular spike trains, near 1 for completely random or Poisson-like activity and becomes larger than 1 for clumped or bursting activity. By convention, neurons whose CV falls in the lowest 1/3 of a sample of vestibular afferents are deemed “regular,” neurons whose CV falls in the largest 1/3 are deemed “irregular,” while neurons with intermediate CV are deemed “intermediate” (4, 10). As Goldberg (12) reiterated, this convention is a rhetorical convenience with no empirical basis.

#### 2.2.2 Candidate models

The selected records are observations from a stationary renewal process that can be modeled as samples from a constant-parameter probability distribution of interval lengths. This is a complete model because the event times themselves, up to an arbitrary start time, can be recovered from the sequence of intervals between them. Since interval lengths must be positive and can have arbitrary length any candidate model must be probability density functions *f*(*t*; α) defined on *t*>0 with parameters α.

Previous studies have shown a consistent pattern of ISI distributions in vestibular afferent spike trains. ISI distributions of the most regular afferents have narrow distributions which are nearly symmetrical and approximately Gaussian, with standard deviations much smaller than mean interval length (σ≪μ). A Gaussian with σ≪μ>0 has essentially no probability mass below zero and can be treated as a density on *t*>0. ISI distributions of more irregular neurons tend to be more right-skewed with larger CVs, while interval distributions of the most irregular neurons resemble exponential distributions, with standard deviation similar to mean interval length (CV = 1). Suitable candidate models therefore are positive-valued, continuously-parameterized probability densities whose shape transforms continuously between limiting cases resembling Gaussian and Exponential distributions.

Candidate models are presented in three groups, to provide transparency about how we selected these candidates and were ultimately led by analysis of the data in three successive stages, to obtain a model that accurately reproduces the shape of the data distribution and quantifies the information that it contains. The first group (1.1–1.5) is the initial set of candidates chosen because they represent models of simple physical processes that are at least somewhat analogous to the canonical “noisy integrate-and-fire” model of a stochastic neuron and/or have been applied previously to model spiking statistics of neurons, including vestibular semicircular canal afferent neurons. The second group (2.1–2.3) contains modified forms of candidates from the first group, which appeared promising after the first round of fitting. These models have an additional parameter which, as explained below, we expected would provide a better fit. To construct the third group of candidates (3.1–3.3) we modified the same group of “promising” candidates by incorporating the additional parameter in a different way, for reasons that will be explained.

Candidate 1.1: *Weibull*


$$f_{WB}\left(t;\;\lambda ,\kappa \right)=\{\begin{array}{c} \frac{\kappa }{\lambda }{\left(\frac{t}{\lambda }\right)}^{\kappa -1}e^{-{\left(\frac{t}{\lambda }\right)}^{k}}\;t\geq 0\\ 0\;t<0 \end{array}$$

is the distribution of intervals between events when event rate is proportional to a power of the waiting time since the last event. This is a birth-death model with “aging.” When κ = 1 (constant event rate) the Weibull reduces to an Exponential distribution.

Candidate 1.2: *Log-normal*


$$\begin{array}{l} f_{LN}\left(t;\;\mu ,\sigma \right)=\;\frac{1}{t\sigma \sqrt{2\pi }}e^{\left(-\frac{{\left(\ln\;t-\mu \right)}^{2}}{{2\sigma }^{2}}\right)} \end{array}$$

is the distribution of outcomes of a growth process involving multiplicative interactions among many small random effects. Multiplicative interactions are additive on a log scale, so the log of the outcome has a Gaussian or normal distribution because of the Central Limit Theorem.

Candidate 1.3: *Erlang*


$$\begin{array}{l} f_{ERL}\left(t;\;\kappa ,\mu \right)=\frac{t^{\kappa -1}e^{\frac{-t}{\mu }}}{\mu^{\kappa }\left(\kappa -1\right)!} \end{array}$$

where the *shape parameter*, κ, is a positive integer and the *scale parameter*, μ, is a positive real number, is the distribution of waiting times for κ events in a Poisson process when the average waiting time is μ (such that the average waiting time in the underlying Poisson process is $\frac{\mu }{\kappa }$). When κ = 1 the Erlang reduces to an Exponential distribution, the waiting time distribution for events in a Poisson process. This has been a popular model of neuronal firing variability, including for vestibular afferent neurons, because of its flexible shape which resembles empirical interval distributions, and because it has a simple mechanistic interpretation as the waiting time for the accumulation of quantal events occurring at random times to reach a threshold (25, 26).

Candidate 1.4: *Birnbaum-Saunders or Cumulative Damage*


$$\begin{array}{l} f_{BBS}\left(t;\;\beta ,\gamma \right)=\;\frac{\sqrt{\frac{t}{\beta }}+\sqrt{\frac{\beta }{t}}}{2\gamma t\sqrt{2\pi }}e^{\left(-\frac{{\left(\sqrt{\frac{t}{\beta }}-\sqrt{\frac{\beta }{t}}\;\right)}^{2}}{{2\gamma }^{2}}\right)} \end{array}$$

is the distribution of waiting time for the accumulation of events with a Gaussian distribution of amplitudes occurring at random times to reach a threshold. It is also known as the Cumulative Damage distribution because of its application to modeling time-to-failure of a system subjected to impacts with random magnitudes occurring at random times. It is a physically plausible model of time to threshold for a neuron receiving EPSPs with Gaussian amplitudes, which fits spike train data from real neurons and biophysically realistic computational neural models (27).

Candidate 1.5: *Inverse Gaussian or Wald*


$$\begin{array}{l} f_{WLD}\left(t;\;\mu ,\lambda \right)=\sqrt{\;\frac{\lambda }{2\pi t^{3}}}e^{\left(-\frac{\lambda {\left(t-\mu \right)}^{2}}{{2\mu }^{2}t}\right)} \end{array}$$

is the distribution of waiting times for Gaussian noise with mean 1/μ with and variance 1/λ to integrate to a threshold at 1. It models the first passage time (time to reach a barrier or integrate to a threshold) of a drift-diffusion process, i.e., Brownian motion in constant flow (28, 29).

The second candidate group was constructed by modifying candidates selected from Group 1 by adding a time offset or delay term to each model. The reason for adding this term is explained in the results section, following analysis of the fitted Group 1 models.

Candidate 2.1: *Offset Erlang*


$$\begin{array}{l} f_{OEL}\left(t;\;\kappa ,\mu ,\;\tau \right)=\tau +\frac{t^{\kappa -1}e^{\frac{-t}{\mu }}}{\mu^{\kappa }\left(\kappa -1\right)!} \end{array}$$

Candidate 2.2: *Offset Wald*


$$\begin{array}{l} f_{OWL}\left(t;\;\mu ,\lambda ,\tau \right)=\tau +\sqrt{\;\frac{\lambda }{2\pi t^{3}}}e^{\left(-\frac{\lambda {\left(t-\mu \right)}^{2}}{{2\mu }^{2}t}\right)} \end{array}$$

Candidate 2.3: *Offset Birnbaum-Saunders*


$$\begin{array}{l} f_{OBS}\left(t;\;\beta ,\gamma ,\tau \right)=\;\tau +\frac{\sqrt{\frac{t}{\beta }}+\sqrt{\frac{\beta }{t}}}{2\gamma t\sqrt{2\pi }}e^{\left(-\frac{{\left(\sqrt{\frac{t}{\beta }}-\sqrt{\frac{\beta }{t}}\;\right)}^{2}}{{2\gamma }^{2}}\right)} \end{array}$$

The third candidate group was constructed by replacing the constant offset parameter τ in the Group 2 models with an Exponentially distributed random time offset having mean τ. In each case this creates a new random variable as the sum of two random variables, whose distribution is the convolution of the distributions of the components. We changed the time offset from a fixed value to a random value for reasons explained in the results section following analysis of the fitted Group 2 models.

Candidate 3.1: *Exerlang*


$$\begin{array}{l} f_{EXE}\left(x;\;\kappa ,\mu ,\;\tau \right)={\frac{1}{\tau {\left(1-\frac{\mu }{\tau }\right)}^{\kappa }}e}^{-\frac{x}{\tau }}\;gammainc\;\left(x\left(\frac{1}{\mu }-\frac{1}{\tau }\right),\;\kappa \right) \end{array}$$

This expression for the convolution of an Exponential distribution and an Erlang distribution was obtained using *Mathematica* (Wolfram Research, Illinois, USA). *Gammainc* is the MATLAB incomplete gamma function, a MATLAB built-in special function. The incomplete gamma function is defined slightly differently in MATLAB and *Mathematica*, so the result derived by *Mathematica* requires adjustment to obtain the formula given above.

Candidate 3.2: *Exwald*


$$f_{EXW}\left(x;\;\mu ,\lambda ,\;\tau \right)=\{\begin{array}{c} e^{\left(\frac{\lambda }{\mu }-\frac{t}{\tau }\right)}\frac{\left(\frac{\left(erfc\left(b-c\right)\right)}{d+d\left(erfc\left(b+c\right)\right)}\right)}{\left(2\tau \right)}\;if\;a\geq 0\\ \frac{e^{\left(\frac{\lambda }{\mu }-\frac{t}{\tau }\right)}e^{-\left(b^{2}+at\right)}Re\left(w\left(\sqrt{-at+ib}\right)\right)}{\tau }\;otherwise. \end{array}$$

Where $a=\frac{\lambda }{\left(2\mu^{2}\right)}-\frac{1}{\tau }$, $b=\sqrt{\frac{\lambda }{\left(2t\right)}}$ and $c=\sqrt{at}$. erfc is the complementary error function, *w* is the Fadeeva scaled complex complementary error function (30), $i=\;\sqrt{-1}$ and *Re*(*z*) is the real part of the complex number *z*. This expression was modified from formulae given by Schwarz (31), by setting the barrier distance/threshold level parameter in the Wald component of Schwarz's derivation to 1 and scaling the other parameters accordingly. We found that this expression can be numerically unstable when λ≪μ (diffusion negligible compared to drift) or τ≪μ (Exponential component negligible compared to Wald component). In the former case we reduced the Wald drift-diffusion component to a pure drift, approximating the Exwald using an Exponential distribution with fixed time offset, μ. In the latter case we removed the Exponential component, approximating the Exwald using only the Wald component. None of our data were fitted by models with parameters in regions of parameter space where these approximations were applied, but it was necessary to include these approximations to prevent numerical instability in the numerical search procedure used for fitting the models, which explores a wider parameter space before converging.

Candidate 3.3: *Exgaussian*


$$\begin{array}{l} f_{EXG}\left(x;\;\mu ,\sigma ,\tau \right)=\frac{1}{2\tau }e^{\left(\frac{2\left(\mu -x\right)+\sigma^{2}}{\tau }\right)}erfc\left(\mu -x+\frac{\sigma^{2}}{\tau }\right) \end{array}$$

This expression for the convolution of a Gaussian distribution with mean μ and variance σ^2^ and an Exponential distribution with mean interval parameter τ was derived analytically using *Mathematica* (Wolfram Research, Illinois, USA). In this expression, $erfc\left(x\right)\;=\;\frac{2}{\sqrt{\pi }}\overset{\infty }{\underset{x}{\int }}e^{-t^{2}}dt$, is the complementary error function, a MATLAB built-in Special Function.

#### 2.2.3 Fitting and model selection criteria

Given an observed probability distribution *p*(*t*), and a model *q*(*t*), the Kullback-Liebler divergence from *q*(*t*) to *p*(*t*), also known as entropy of *p*(*t*) relative to *q*(*t*), is


(1) $$\begin{array}{l} D_{KL}\left(p||q\;\right)=\int p\left(t\right)\log_{2}\frac{p\left(t\right)}{q\left(t\right)}dt. \end{array}$$

*D*_*KL*_ measures information lost in bits when *q*(*t*) is used to approximate the empirical distribution, *p*(*t*). Given a set of candidate models, minimum *D*_*KL*_ identifies the candidate that minimizes the expected information in future observations, given what has been observed (3, 32, 33). This criterion, which avoids the problem of over-fitting (i.e., models with too many free parameters that fit better but make worse predictions) provided the theoretical foundation used by Akaike (34) to derive his famous information criterion (AIC) for model selection (n.b. the cited paper is a reprint of Akaike's original 1973 conference paper). AIC is asymptotically equivalent to *D*_*KL*_, i.e., is expected to give the same answer as *D*_*KL*_ on average in the long run. AIC is usually used instead of *D*_*KL*_ because *D*_*KL*_ requires the true data distribution to be known. We apply *D*_*KL*_ under the bootstrap assumption that our data sets are large enough represent the true shapes of the distributions that they are drawn from, which is justified by inspection of ISI histograms (35, 36).

Given *N* observations *t*_1_, *t*_2_, ⋯ , *t*_*N*_, the empirical distribution can be represented as a normalized frequency histogram, with probability $p_{k}=\frac{n_{k}}{N}$ in the *k* th bin, where *n*_*k*_ is the number of observations in the *k* th bin. Assuming that *q*(*t*)≈*q*_*k*_ is constant in the kth bin, the expression for *D*_*KL*_ reduces to a sum,


(2) $$D_{KL}\left(p\|q\;\right)=\int p\left(t\right)\log_{2}\frac{p\left(t\right)}{q\left(t\right)}dt=\;\sum p_{k}\log_{2}\frac{p_{k}}{q_{k}}$$

If each bin is very narrow and contains at most one observation then *q*(*t*) = *q*_*k*_ and the normalized histogram reduces to a particle model, with probability $p\left(t_{k}\right)=\frac{1}{N}$ at the observed points *t*_*k*_ and zero elsewhere. In that case the expression for *D*_*KL*_ reduces to


(3) $$\begin{array}{l} D_{KL}\left(p\|q\;\right)=\int \frac{\delta \left(t-t_{k}\right)}{N}\log_{2}\left(\frac{\delta \left(t-t_{k}\right)}{N\;q\left(t\right)}\right)dt\\ \;=-\frac{1}{N}\sum \log_{2}\left(q\left(t_{k}\right)/N\right). \end{array}$$

Thus


(4) $$\begin{array}{l} D_{KL}\left(p||q\;\right)=-\frac{1}{N}\sum \log_{2}\left(q\left(t_{k}\right)\right)+\;\log_{2}\left(N\right)\;, \end{array}$$

is negative log-likelihood with a logarithmic penalty on sample size.

Since the sample size is fixed in each record, fitting a model by minimum *D*_*KL*_ is equivalent to fitting a model by maximum likelihood for any given neuron. However, across neurons KLD scales the log-likelihood by the entropy of the empirical distribution, giving a measure of model performance which is independent of differences in variability of spike time data from different neurons. For example, regular neurons have narrow ISI distributions with high probability densities and generate more spikes during the 20-second recording period because they fire faster. As a result, the likelihood for any given model is generally larger for more regular neurons, and using maximum likelihood would bias selection in favor of candidates that are better at fitting regular neurons. *D*_*KL*_ avoids this problem. Having said that, we found that using maximum likelihood as a model-selection criterion leads to qualitatively similar results as using *D*_*KL*_, and does not affect our conclusions.

#### 2.2.4 Model fitting

Models were fitted using the MATLAB function fminseachbnd 1.4.0 which implements the Nelder-Mead simplex algorithm (37) with constraints or bounds on allowable parameter values. The constraints were applied to prevent the algorithm from stepping outside the region of parameter space in which a model is defined (e.g., negative mean interval length), which would produce meaningless results and/or numerical instability.

#### 2.2.5 Analysis of fitted models

Candidate models have at most 3 parameters meaning that fitted parameters for each neuron can be visualized as a point in 3D, and parameters fitted to all records form a cloud in 3D space. The cloud of points fitted to our data is roughly ellipsoidal in log-log axes. We computed the major axes of this ellipsoid using the *pca* function in the MATLAB Statistics Toolbox. We computed the convex hull of parameter estimates in 2D projections (the smallest polygon enclosing all points) using the MATLAB built-in function convhull. We used the first principal component axis to generate curves in parameter space showing the predicted value of a parameter given some other parameter. For example, to show how a model parameter α relates to the summary statistic CV= $\frac{s}{\bar{x}}$, we find parameters on the first principal component axis corresponding to a model with this CV. Simple closed expressions can be found in all cases, i.e., it is not necessary to use numerical optimization/search procedures to compute these curves.

## 3 Results

### 3.1 Summary statistics

**Figure 1:**
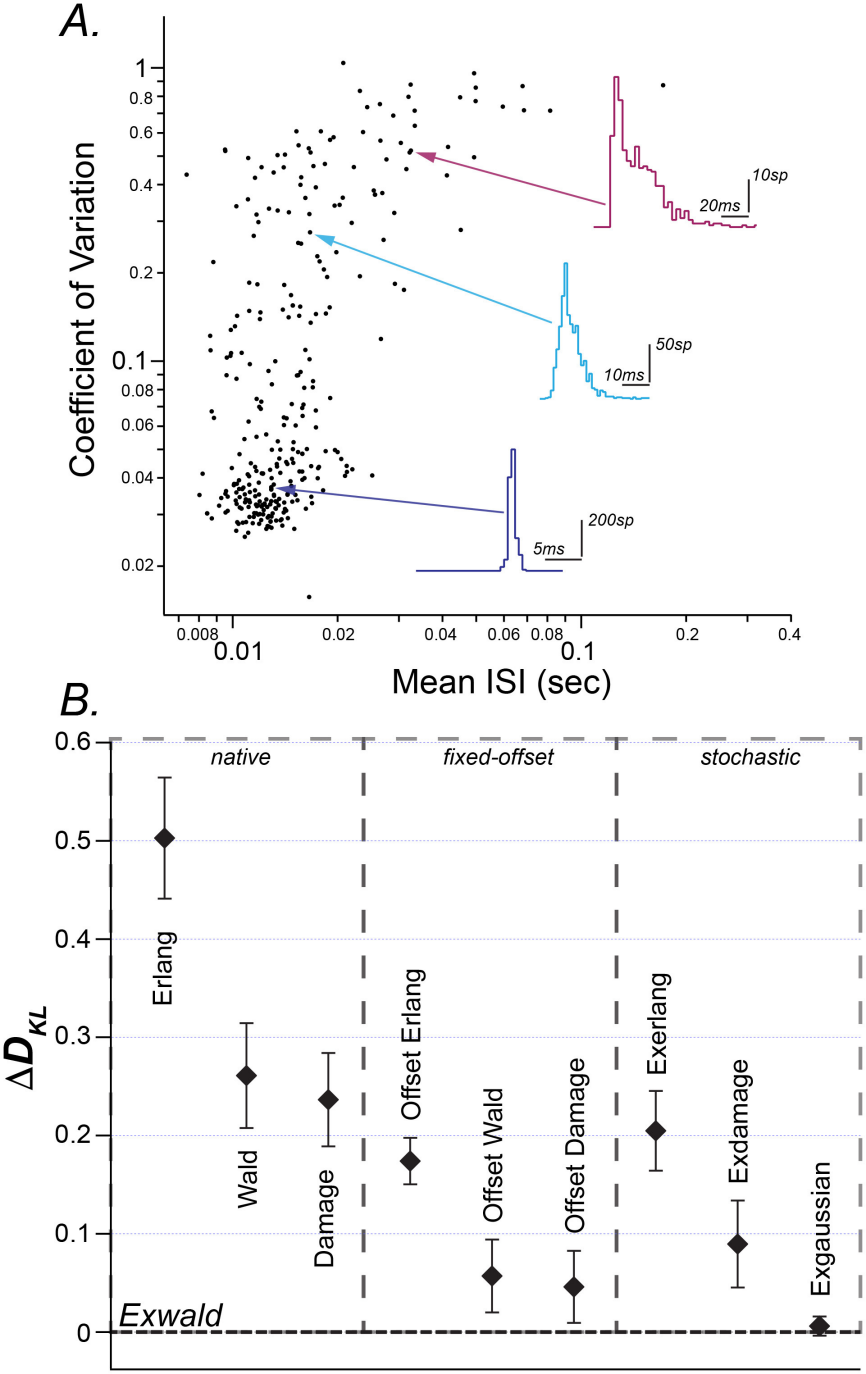
**(A)** Mean interspike interval (ISI) vs. coefficient of variation (CV) of spontaneous discharge for 306 horizontal semicircular canal afferent neurons (filled circles). Representative ISI histograms are shown for regular (blue), intermediate (teal), and irregular (magenta) afferents with arrows indicating the location of the selected neuron on the scatterplot. Aspect ratios of the histograms are different (note scale bars). **(B)** Kullback-Liebler (K-L) divergence of fitted candidate models subtracted from the Kullback-Liebler divergence of the best fitted candidate (Exwald). Candidates are grouped into random walk models (native), fixed offset random walk models (fixed-offset) and stochastic random walk models (stochastic). Each point (black diamond) is the mean K-L divergence for a given model over all spike trains. The error bars represent standard errors of these means.

Figure 1A is a scatterplot of conventional ISI summary statistics, mean and coefficient of variation. It shows the heterogeneity of spontaneous discharge characteristics, and the tendency for neurons with shorter mean intervals (higher firing rates) to have more regular firing patterns. The average mean interval is 16.9 ms (± 13.0 ms) and the average CV is 0.17 (± 0.22). This plot closely resembles scatterplots of mean ISI vs. CV in previous reports of vestibular afferent neuron spiking activity [c.f. Baird et al. (24), Figure 1; Goldberg (12); Honrubia et al. (38), Figure 6B; Hullar et al. (39), Figure 1]. The scatterplot shows the wide variation in mean interval length and CV with no indication of distinct groups within the population.

ISI histograms for three selected afferents are overlaid on the scatterplot. They closely resemble ISI distributions previously reported in vestibular afferents in various species (3). The inset shows these three distributions plotted on common axes. This illustrates that while mean and CV reveal substantial diversity in spontaneous behavior of these neurons, these descriptive statistics fail to characterize the shapes of ISI distributions and the large, systematic shape changes across the population. Regular afferents, with faster mean firing rates tend to have narrow, approximately Gaussian ISI distributions, while irregular, slower-firing afferents tend to have positively skewed ISI distributions. The most irregular afferents, with CVs near 1, have ISI distributions that resemble right-shifted or left-censored Exponential distributions. Exponential interval distributions are characteristic of Poisson processes, for which the average time between events is fixed but event times are random (40, 41). Poisson distributions have the unique property that removing intervals shorter than some specified duration (left-censoring) is equivalent to right-shifting the distribution by that duration.

### 3.2 Fitted models

Group 1 candidate models (Weibull, Log-normal, Erlang or Integer Gamma, Inverse Gaussian or Wald, and Birnbaum-Saunders or Cumulative Damage Distribution; see Methods) are continuous probability distributions defined on positive intervals. For brevity, we refer to the Birnbaum-Saunders/Cumulative Damage Distribution as the Damage distribution. These candidates were selected because they possess the requisite property of having Gaussian-like shapes for some parameter values and highly skewed Exponential-like shapes for other parameter values. Weibull and Lognormal candidates were quickly eliminated because they fit poorly and there are obvious qualitative discrepancies between the shapes of the empirical distributions and these candidate models.

The remaining candidates, Erlang, Wald, and Damage distributions, all seem capable of generating the shapes of the empirical interval distributions. In addition, they are all waiting time distributions for random counting or integrating processes to reach a threshold and can be interpreted in terms of simple models of physical mechanisms that underlie neuronal spiking. All have previously been proposed as models of neuronal spiking variability (See Methods). Each of these distributions has two free parameters.

The relative goodness of fit for these three models is shown in the left column of Figure 1B (*native*). The vertical axis in this figure (Δ*D*_*KL*_) is the mean difference between Kullback-Leibler Divergence from model to data for each model relative to the Kullback-Leibler Divergence of the best-fitting model (See *Methods*). Error bars represent the standard error of mean Δ*D*_*KL*_. By this criterion the Damage distribution is the best candidate, followed by the Wald and the Erlang.

Inspection of plots of best-fitting models overlaid on the empirical interval distributions showed that in many cases a fitted model deviated systematically from the data, but manual adjustment of parameters indicated that the model should be capable of fitting the shape of the empirical distribution much more accurately than it did. We hypothesized that this may be because the parameters of these models do not affect shape and location independently. Changing the value of a parameter generally causes a change in the shape of the distribution and to shift the distribution along the time axis. Because the Kullback-Liebler criterion harshly penalizes models that assign negligible probability to observed values, minimum *D*_*KL*_ favors models that place probability mass in locations where there is data over models whose shapes match the shapes of the data distributions.

We reasoned that adding a time delay parameter would allow Group 1 models to match the shapes of the data distributions and shift their locations to align with the data. The second panel in Figure 1B (*fixed offset*) shows that this additional offset parameter improves *D*_*KL*_ for each model, and visual inspection of plots confirmed that all three offset models can accurately match the shapes of the empirical distributions in the correct locations. The performance improvement due to the additional free parameter is similar for each model, so that their ranking remains the same. The offset Damage model is the best, followed by the offset Wald and offset Erlang.

Although introducing a time offset parameter confirmed that there is (at least) a degree of freedom missing in each of the group 1 (*native*) statistical models, a pure time offset in a model of neuronal spiking is implausible. This is not only because if interpreted realistically this term would represent a biophysical mechanism capable of producing precisely timed fixed intervals, with different durations in different neurons, but also because some of the fitted time offset parameters in the group 2 models are negative. Negative time offsets would require a clock that starts at a fixed time before a random future event, which would violate causality.

The simplest realistic way to extend the group 1 models in a way that adds a degree of freedom in location is to include a Poisson process in series. A Poisson process has only one parameter, the mean interval length, and has maximum entropy given the constraint that intervals must have positive real durations with a finite mean (66). It follows that the modification can be justified by Jaynes' Principle of Maximum Entropy (32) because it incorporates what was learned from fitting the Group 1 (*native*) candidate models with no additional assumptions or constraints.

Intervals between events in a Poisson process have an Exponential distribution. The third panel of Figure 1B (*stochastic*) shows that adding an Exponentially distributed random delay term to each of the Erlang, Damage and Wald models improves the fit of all these models. As might be expected, since the time-offset models fit quite precisely and the Poisson series element must introduce a shape change in addition to a time offset, the Poisson element doesn't improve the fit of the Erlang or Damage models as much as a pure time offset does. Surprisingly, however, it improves the fit of the Wald model by even more than a pure time offset does. Adding an additional degree of freedom in this way not only allows the model to position itself over the data but also allows it to better match the shape of the data distribution.

An Exponential distribution in series with a Wald distribution is called an Exwald distribution (31, 42). Analogously, we refer to the Exponentially-extended Erlang and Damage distributions the Exerlang and Exdamage distributions respectively.

**Figure 2:**
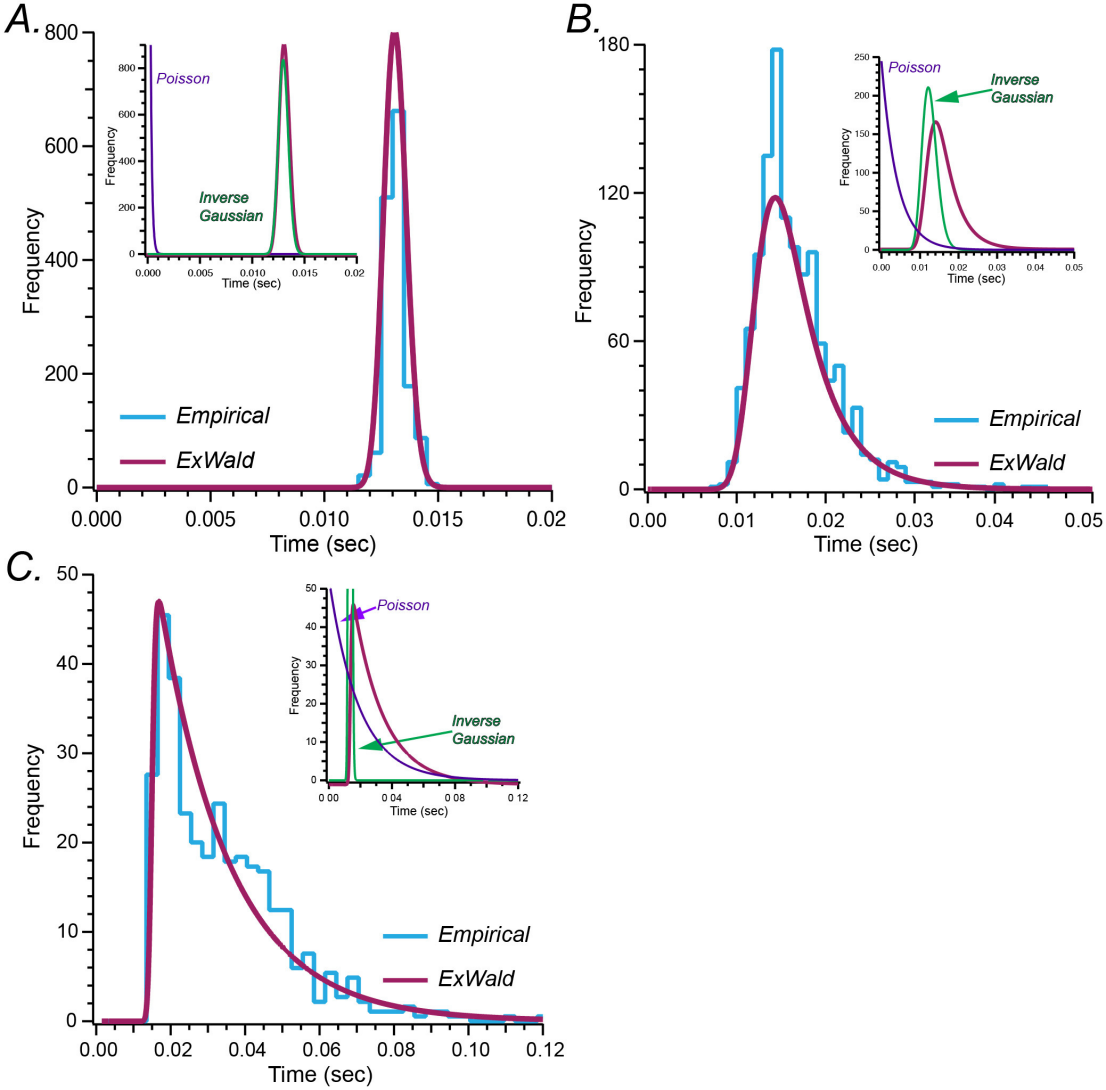
**(A–C)** Fitted Exwald models (magenta) overlaid on empirical ISI histograms (cyan) for the three representative neurons shown in Figure 1A. In each case the inset plots show the decomposition of the Exwald model into its Exponential (or Poisson; purple) and Wald (or Inverse Gaussian; green) components. As in Figure 1A the axes have different scales for each represented afferent.

Figure 2 shows Exwald models fitted to ISI histograms for a regular, an intermediate and an irregular unit. These are the same example units shown in Figure 1A. Components of the intermediate model, for which the decomposition is easiest to see, are labeled. All neurons, not just these three examples, have a refractory period in the order of 10 ms during which the probability of spiking is essentially zero. The refractory period appears to be determined by the Wald component, while the extent of the tail, corresponding to spiking irregularity, appears to be determined by the Exponential or Poisson component. Shape and location parameters of the Wald components are similar for all three neurons, while the parameter of the Poisson component is larger for more irregular neurons.

Figure 2 shows Exwald models fitted to spontaneous discharge ISI histograms for the three units displayed in Figure 1A. Inset plots show the Exponential and Wald components of each fitted model. Wald components tend to be similar for all neurons, Gaussian-like with means around 13 ms and small positive skewness. Differences in shapes of model distributions are mostly a result of changes in the Poisson mean interval parameter. This suggests that it may be possible to replace the Wald component of the Exwald model with a Gaussian distribution to obtain simpler models that fit as well as the Exwald. We tested this possibility by adding an Exponential distribution in series with a Gaussian distribution (Exnormal) to the “stochastic” candidate set. The Exnormal model does not fit the data as well as the Exwald model does, although the difference is small (Figure 1B).

In summary, Figure 1B shows that the Exwald distribution is the best model among candidates that we tested. Figure 2 shows that Exwald distributions can accurately match the shapes and locations of disparate ISI distributions of semicircular canal afferent neuron spontaneous discharge, using only three free parameters.

### 3.3 Analysis of the Exwald model

The Exwald is the distribution of intervals generated by an Inverse Gaussian process in series with a Poisson process. Each interval in an Exwald distribution is the sum of an interval drawn from the Wald component and an interval drawn from the Exponential component. The Exwald has three parameters: μ and λ, which are the mean interval and shape parameters of the Wald distribution, and τ, which is the parameter of the Exponential interval distribution of the Poisson process. The parameters are all positive quantities with dimensions of time, reported here in milliseconds.

**Figure 3:**
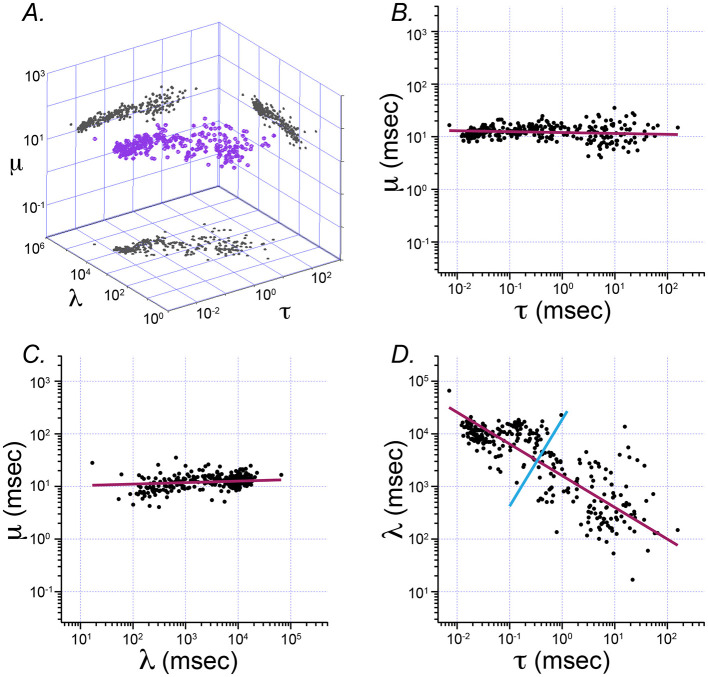
Principal component analysis of Exwald model parameters. **(A)** The 3D cloud of fitted parameter values (purple) projected into each of the coordinate planes on log-log axes. The aspect ratio is the same on all axes, such that each grid unit represents a tenfold change in magnitude for any of the parameters. **(B–D)** Projection planes showing relationships between each pair of parameters (**B**: τ vs. μ, **C**: l vs. μ, and **D**: τ vs. l). Magenta lines represent the first principal component axis projected onto the 2D parameter planes in each case. The cyan line in D represents the second principal component for that distribution. These plots show that almost all variation in parameter space (and thus in ISI shape space) is explained by τ and l. These parameters both vary over several orders of magnitude. In contrast, μ is similar in all models, close to the average value of 12.7 ms.

Figure 3 shows the result of principal component analysis (PCA) of Exwald model parameters. The fitted parameters form a flattened, elongated ellipsoidal cloud of points when plotted on log-log axes in 3D. PCA was used to find the major axes of an ellipsoid fitted to this cloud. The four panels show the parameter cloud and the principal component axes projected into the three coordinate planes of the parameter space.

Figures 3B, C show that the first principal component axis of the parameter distribution is almost parallel to the τ − λ plane, with values of μ clustered around the mean value of 12.7 ms. In contrast, τ varies over roughly 4 orders of magnitude while λ varies over roughly 2 orders of magnitude. Figure 3D shows that most of the variation among parameters, and correspondingly most of the differences between interval distributions, can be explained by only two parameters, τ and λ. The first principal component has a slope near −0.5 in the τ − λ plane. A slope of −0.5 on log-log axes indicates an inverse square relationship between these parameters $\lambda \propto \;\frac{1}{\sqrt{\tau }}$.

#### 3.3.1 Relationship between Exwald model parameters and conventional summary statistics

**Figure 4:**
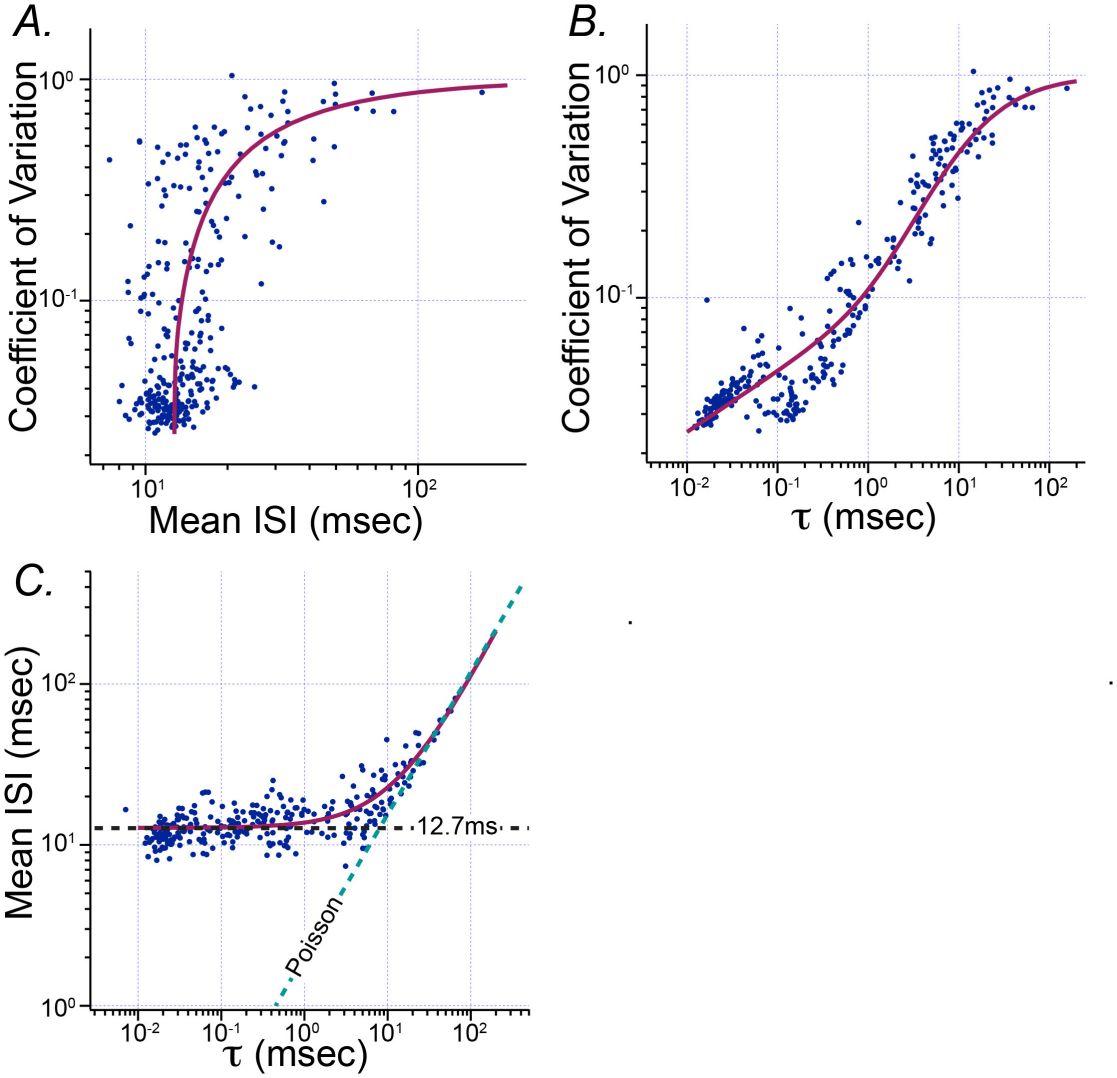
Relationship between the Poisson parameter τ of the Exwald model and conventional summary statistics of spontaneous activity, mean ISI and CV. **(A)** Scatterplot of mean ISI vs. CV (c.f. Figure 1A). The magenta curve drawn over the scattered data points shows CV computed from the Exwald model whose parameters are given by the first principal component of fitted parameter values for a specified mean ISI. **(B)** Scatterplot of τ vs. CV. The magenta curve shows CV computed from the Exwald model on the first principal component axis for specified τ. **(C)** Scatterplot of τ vs. mean ISI. Magenta curve shows the mean ISI computed from the Exwald model on the first principal component axis for a given τ.

Figure 4 shows how the parameters of fitted Exwald models are related to the conventional summary statistics historically used to describe the statistical diversity of vestibular afferent firing patterns, mean ISI and CV. The curve in Figure 4A shows the Exwald model-predicted CV for parameters on the first principal component axis corresponding to a model with the specified mean ISI. It is a projection of PC1 from log(τ)−log(λ) parameter space into mean ISI–CV parameter space. It shows that the Exwald model predicts the known relationship between mean ISI and CV (12, 24). Similarly, the curves in Figures 4B, C show that the Exwald parameter τ is a good predictor of CV and mean ISI.

Figure 4 shows that τ characterizes not only the change in mean and variability of ISI distributions over the population, but also the systematic change in shape of the distributions. For small values of $\tau ,\;(\tau \ll \bar{ISI}\approx 12.7ms$), interval length is largely determined by the Wald component, while for large values of $\tau ,\;(\tau \gg \bar{ISI}\approx 12.7ms$), interval length is largely determined by the Poisson component. Thus τ characterizes the continuous diversity of statistical behavior in vestibular afferent neurons from rapidly firing, regular neurons whose interval distributions resemble narrow Gaussians to slowly firing, irregular neurons whose interval distributions resemble right-shifted or (equivalently) left-censored Exponentials. This parameter characterizes afferent diversity in a more natural and informative way than the conventional summary statistics CV or CV^*^ (c.f. Figure 3).

#### 3.3.2 Distribution of Exwald model shapes in model parameter space

**Figure 5:**
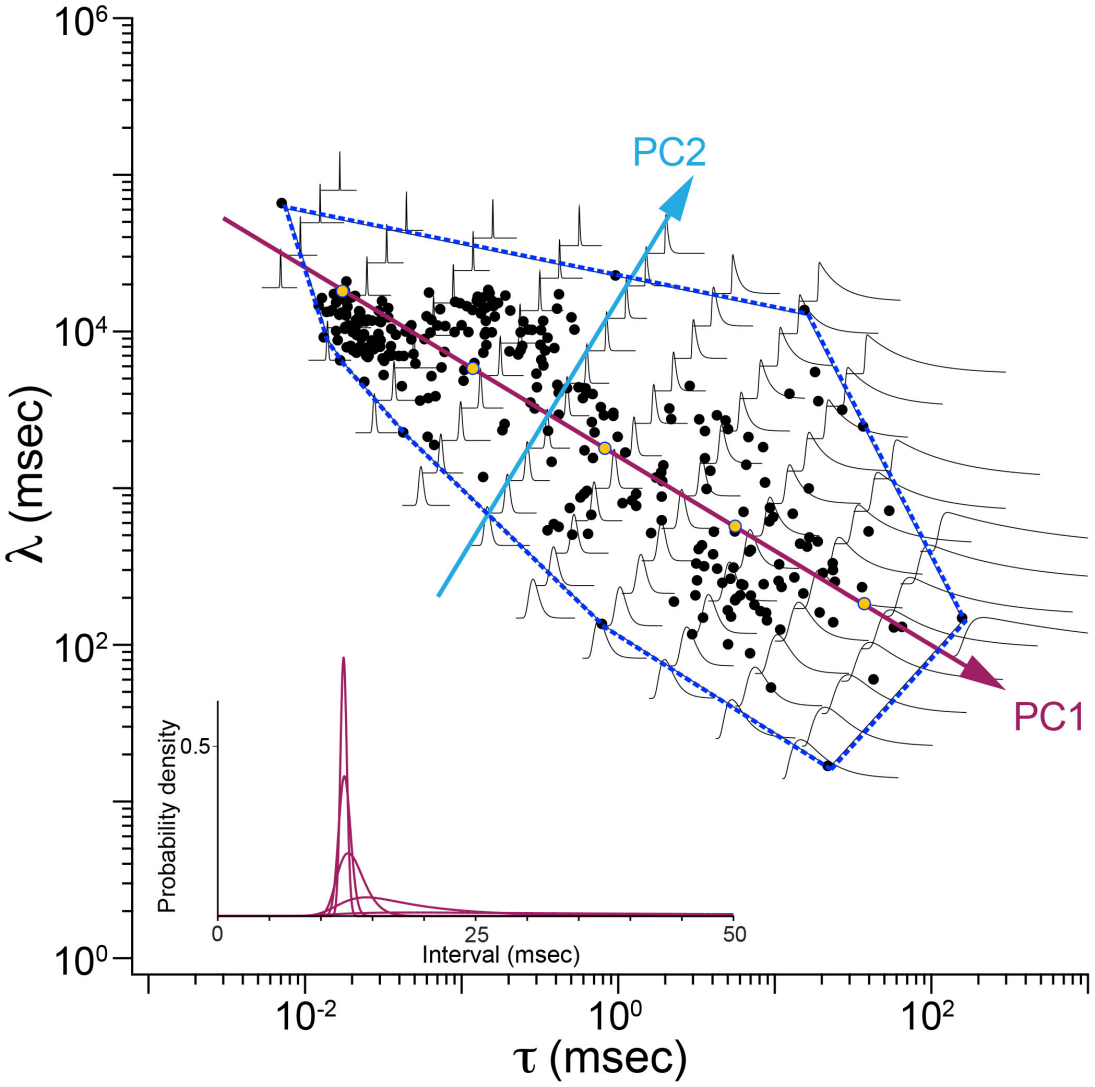
Map of ISI distributions in Exwald parameter space. Scatterplot of fitted parameters in the τ-λ plane (black filled circles), with principal component axes (PC1, PC2) projected onto the plane. The dashed blue line is the convex hull of fitted parameter points. Overlaid Exwald models illustrate how the shape of ISI distributions vary across this region of parameter space. These plots have been scaled so that they have the same height. Inset (lower left) shows the unscaled proportions for five models whose parameters lie on the first principal component axis. These models correspond to the five yellow markers drawn on that axis.

Figure 5 is a map showing how shapes of ISI distributions vary systematically in parameter space. Parameter values fitted to data are plotted as blue disks. The first two principal component axes are shown. The dashed line is the convex hull, the smallest polygon enclosing the fitted parameter points. Shapes of Exwald model interval distributions are drawn on a grid aligned with the principal component axes. For each distribution, t = 0 is plotted at the grid point. The time (horizontal) scales are all the same but the vertical (probability density) axes are scaled so that all distributions have the same peak height. In reality the distributions for the most regular neurons (upper left of the map) are so much taller than the distributions for the most irregular neurons (lower right) that it is infeasible to render the shapes on the same plot without scaling. The inset (lower left) shows the true shapes of five distributions spaced along the first principal component axis.

Figure 5 indicates that the first principal component measures ISI variability, which is strongly predicted by τ (c.f. Figure 4B). The second principal component measures variability of the refractory period, which is strongly predicted by λ, as evidenced by the increasingly steep onset of spiking probability after a refractory period in the direction of the second principal component.

The principal components analysis shows that 91.7% of parameter variance is explained by the first principal component, 7.6% by the second and 0.7% by the third. This indicates that almost all variation in spontaneous discharge behavior of semicircular canal afferent neurons can be explained by a single parameter of the model, corresponding to a single degree of freedom in the underlying mechanisms. Among the Exwald parameters, the Poisson mean interval, τ, is most closely correlated with this degree of freedom. The high proportion of between-neuron variability explained by variation in τ, combined with the fact that CV can be predicted by τ alone (Figures 4B, C), explains why spontaneous firing regularity, quantified by CV or CV^*^, is a useful rhetorical convention for summarizing the behavior of semicircular canal afferent neurons within the diverse population.

## 4 Discussion

### 4.1 Spontaneous spike trains of semicircular canal afferent neurons are refractory-censored Poisson processes

We have shown that diverse spontaneous discharge characteristics of semicircular canal afferent neurons can be accurately quantified by modeling interspike intervals as independent samples from an Exwald distribution. A sample from an Exwald distribution is the sum of two components, a sample from an Exponential distribution and a sample from an Inverse Gaussian distribution. The Exponential is the interval distribution of a Poisson process and the Inverse Gaussian is the distribution of first passage times of a drift-diffusion process. These components can be in any order, but because of a counter-intuitive statistical property of Poisson processes, an Exwald process can be interpreted as a Poisson process with stochastic refractory periods determined by an Inverse Gaussian process. The counter-intuitive property is that the waiting time distribution for the next event in a Poisson process is independent of the time since the previous event (40). If events are blocked for an arbitrary time after an event, then the interval to the next event will be the sum of the blocking time plus a sample from the Poisson process. If the blocking time is a sample from an Inverse Gaussian distribution then the interval will be a sample from an Exwald distribution. A statistician would call this an Inverse Gaussian left-censored Poisson process, while a neuroscientist might refer to it as a Poisson process with stochastic refractory periods determined by an Inverse Gaussian process.

Distributions of refractory periods in fitted models are generally narrow and nearly symmetric with means near $\bar{\mu }=12.7ms$ (Figure 2). In contrast, the parameters of the Poisson component vary over nearly four orders of magnitude, from microseconds to seconds. Consequently, differences in shapes of fitted Exwald models are largely due to differences in the Poisson components. This is surprising because we constructed the Exwald candidate model by adding an Exponential component to the Inverse Gaussian model, after initial fits indicated a need to add a degree of freedom to the location, not the shape, of the Inverse Gaussian model.

### 4.2 A simple ideal physical model can mimic spontaneous activity

Simple physical mechanisms can generate samples from Poisson processes and Wald processes, which can be combined to form a simple mechanism that can generate samples from an Exwald process. A Poisson process can be implemented using a threshold trigger mechanism in noise (43). A Wald process can be implemented by integrating Gaussian noise until the integral reaches a threshold, at which time an event is generated and the integral is reset to its initial level (44). This is familiar to neuroscientists as an integrate-and-fire process. It follows that event sequences whose inter-event intervals are samples from an Exwald process can be generated by a threshold trigger mechanism in noise that is blocked by an integrate-and-fire mechanism after each event.

### 4.3 The ideal physical model is a stochastic dynamical model

As noted in the introduction, continuity suggests that a model of spontaneous activity, the response at a point in head kinematic state space, should be interpretable as a model of dynamical responses in at least a small region near that point. The ideal physical model can be used to demonstrate how the Exwald model could be extended to form a stochastic dynamical model of semicircular canal afferent neuron behavior during head movement.

Suppose that each component of the physical model receives the same noise input, and the mean input level is determined by head angular acceleration around the canal axis. For any fixed mean input level (constant angular acceleration) this model will produce an event sequence with an Exwald interval distribution. Each interval has two components, a Poisson component whose mean rate depends on the mean of the input (head angular acceleration) and an Inverse Gaussian component whose mean rate depends on the integral of the mean of the input (head angular velocity). Poisson processes have highly variable interval lengths (CV = 1) while Inverse Gaussian processes, in the region of parameter space occupied by our fitted models (Figure 5), have much more regular interval lengths (CV = μ/λ).

This simple model predicts the pattern of changes in variability (CV) of semicircular canal afferent neuron discharge during constant angular acceleration (3, 12). It also predicts that irregular neurons (with larger values of τ, which determines the relative contribution of the Poisson component to interval length) tend to fire in phase with head angular acceleration while regular neurons (whose spontaneous interval lengths are dominated by the Inverse Gaussian component) tend to fire in phase with head angular velocity. It remains to be determined whether this model can mimic the stochastic dynamical responses of semicircular canal afferent neuron discharge during arbitrary naturalistic head motion.

### 4.4 There is a unimodal distribution of model parameters across the population

Our data and analysis confirm that “regular” and “irregular” represent two ends of a unimodal distribution of statistical and dynamical characteristics of semicircular canal afferent neurons (3, 12). The Exwald model can explain the co-variation of statistical and dynamical parameters in terms of the relative contribution of regular velocity-sensitive and irregular acceleration-sensitive components that contribute to the response of each afferent. There are not two types of neurons in this population, just a single type with two components. There is no sign of bimodality in the distribution of parameters fitted to our data.

### 4.5 Semicircular canal afferents provide a fast, efficient communication channel from molecular mechanoreceptors to the brain

Mechanoreceptor channels in hair cells in the sensory epithelium of a semicircular canal open when the gating force exceeds a threshold. The gating force depends on the rotational component of the inertial force due to head acceleration around the canal axis plus random fluctuations due to thermal noise (i.e., random molecular motion) in the coupling and transduction mechanisms. Gating energies are so small that thermal noise causes the channels to open and close randomly thousands of times per second even when the head is not moving. These mechanically gated channels are thereby threshold trigger mechanisms whose inputs are noisy observations of head angular acceleration. They gather information about head angular acceleration with sub-thermal noise sensitivity, and with bandwidth exceeding 10 KHz (45–50).

The high energy cost of high average firing rates constrains the bandwidth of information transmission by spiking neurons. The cost becomes prohibitive if average intervals become smaller than a few milliseconds. Spiking neurons generally have refractory periods that enforce longer average interspike intervals (51–53). The Exwald model indicates that semicircular canal afferent neuron spike trains are Poisson processes with mean rates exceeding tens of thousands of events per second (mean interval τ < 0.1 ms), censored by random refractory periods a few milliseconds in duration. Because of random refractory censoring, a semicircular canal afferent neuron spike train is a random subsample from an underlying Poisson process whose rate is specified by the Exponential parameter, τ, of the Exwald model.

A Poisson process with a mean rate in the order of 10 KHz (mean interval 0.1 ms) censored with a mean refractory period in the order of 10 ms will be randomly decimated by a factor of about 100. Statistically independent subsampling in different neurons allows a population containing hundreds of neurons to transmit all of the information contained in a Poisson process whose mean rate greatly exceeds 100 Hz, without paying the punitive energy costs entailed by individual neurons firing at such high rates (54–56). Thus it would be possible for the approximately 1,000 semicircular canal afferent neurons that project from each canal (57, 58) to quickly and efficiently transmit all of the information captured by mechanoreceptor gate-opening events in hair cells to the brain.

### 4.6 Head kinematic state can be inferred from Exwald-distributed observations

The Bayesian posterior distribution of the parameters of a stochastic process can be inferred from a sequence of observations by applying Bayes rule to update a prior estimate of the distribution when each observation is made (59). This is called Bayesian filtering. The Bayesian update rule requires a likelihood function, which specifies the probability distribution of observations as a function of the unknown parameters, and a dynamical model of the observed system.

Our data and analysis suggest that interspike intervals of semicircular canal afferent neuron spike trains can be modeled as observations of head kinematic state, by re-parameterizing the Exwald model in terms of the state variables. The ideal physical model described in Sections 4.2 and 4.3 shows explicitly how this may be done. The model defines a likelihood function for the state variables given each observation. This is because over a small interval the trajectory of any dynamical system can be predicted by taking the tangent to its trajectory at the current state estimate and using a Gaussian with increasing variance to model the growth of uncertainty about the true state as the prediction interval increases. The linear-Gaussian prediction rule is a model of a physical drift-diffusion process (and vice versa), in which the tangent direction and speed corresponds to mean particle velocity (the drift vector) and the increasing variance corresponds to diffusion away from the mean particle position. This principle underlies the Kalman filter and modern Bayesian filtering methods for non-linear dynamical state estimation (60).

We speculate that Bayesian particle filters (59, 61) may provide realistic models of neural computation for Bayesian inference in the brain. A particle filter represents a probability distribution by a random sample of points (particles) drawn from it. These algorithms can be applied to model neural computation by interpreting neural networks in the brain as maps of relevant parameter spaces in which neurons correspond to points in the space and spikes represent particles created at and moving between these points (62–65).

## Data Availability

The raw data supporting the conclusions of this article will be made available by the authors, without undue reservation.
